# Short-term effectiveness and side effects of ketogenic diet for drug-resistant epilepsy in children with genetic epilepsy syndromes

**DOI:** 10.3389/fneur.2024.1484752

**Published:** 2024-09-18

**Authors:** Osama Y. Muthaffar, Anas S. Alyazidi, Daad Alsowat, Abdulaziz A. Alasiri, Raidah Albaradie, Lamyaa A. Jad, Husam Kayyali, Mohammed M. S. Jan, Ahmed K. Bamaga, Mohammed A. Alsubaie, Rawan Daghistani, Saleh S. Baeesa, Meshari A. Alaifan, Abdelhakim Makraz, Abrar N. Alsharief, Muhammad Imran Naseer

**Affiliations:** ^1^Pediatric Neurology Division, Department of Pediatrics, Faculty of Medicine, King Abdulaziz University, Jeddah, Saudi Arabia; ^2^Department of Pediatrics, Faculty of Medicine, King Abdulaziz University, Jeddah, Saudi Arabia; ^3^Epilepsy Integrated Practice Unit, Neuroscience Center, King Faisal Specialist Hospital and Research Centre, Riyadh, Saudi Arabia; ^4^Neuroscience Center, King Fahad Specialist Hospital, Dammam, Saudi Arabia; ^5^Pediatric Neurology Department, National Neuroscience Institute, King Fahad Medical City, Riyadh, Saudi Arabia; ^6^Department of Pediatrics, Neurology Division, Sidra Medicine, Doha, Qatar; ^7^Department of Pediatrics, King Faisal Specialist Hospital & Research Centre, Jeddah, Saudi Arabia; ^8^Department of Neuroscience, King Faisal Specialist Hospital and Research Center, Jeddah, Saudi Arabia; ^9^Center of Excellence in Genomic Medicine Research (CEGMR), King Abdulaziz University, Jeddah, Saudi Arabia; ^10^Department of Medical Laboratory Technology, Faculty of Applied Medical Sciences, King Abdulaziz University, Jeddah, Saudi Arabia

**Keywords:** drug-resistant epilepsy, ketogenic diet, epilepsy syndromes, dietary therapies, children, seizure

## Abstract

**Background:**

Drug-resistant epilepsy (DRE) impacts a significant portion, one-third, of individuals diagnosed with epilepsy. In such cases, exploring non-pharmacological interventions are crucial, with the ketogenic diet (KD) standing out as a valuable option. KD, a high-fat and low-carb dietary approach with roots dating back to the 1920s for managing DRE, triggers the formation of ketone bodies and modifies biochemistry to aid in seizure control. Recent studies have increasingly supported the efficacy of KD in addressing DRE, showcasing positive outcomes. Furthermore, while more research is needed, limited data suggests that KD May also be beneficial for specific genetic epilepsy syndromes (GESs).

**Objective:**

This study aimed to assess the short-term efficacy of KD among pediatric patients diagnosed with GESs.

**Materials and methods:**

This is a multi-center retrospective analysis of pediatric patients with GESs diagnosed using next-generation sequencing. The enrolled patients followed the keto-clinic protocol, and the KD efficacy was evaluated at 3, 6, and 12-month intervals based on seizure control and compliance. The collection instrument included demographic, baseline, and prognostic data. The collected data was coded and analyzed promptly.

**Results:**

We enrolled a cohort of 77 patients with a mean current age of 7.94 ± 3.83 years. The mean age of seizure onset was 15.5 months. Notably, patients experienced seizures at a younger age tended to have less positive response to diet. Overall, 55 patients responded favorably to the diet (71.4%) while 22 patients (28.6%) showed no improvement. Patients with genetic etiology showed a significantly more favorable responses to the dietary intervention. Patients with Lennox–Gastaut syndrome showed the most significant improvement (14/15) followed by patients with Dravet syndrome (6/8), and West syndrome (3/4). The number of used anti-seizure medications also played a significant role in determining their response to the diet. While some patients experienced mild adverse events, the most common being constipation, these occurrences were not serious enough to necessitate discontinuation of the diet.

**Conclusion:**

The study revealed a high improvement rate in seizure control, especially among younger patients and those with later seizure onset. The success of dietary treatment hinges greatly on early intervention and the patient’s age. Certain genetic mutations responded favorably to the KD, while efficacy varied among various genetic profiles.

## Introduction

Drug-resistant epilepsy (DRE) can constitute a third of all patients with epilepsy (1, 2), and it has been defined as the failure of adequate trials of two tolerated, appropriately chosen and used anti-seizure medication (ASM) schedules as monotherapy or combined therapy to achieve sustained control of seizures (3). In children with DRE, prompt referral to specialized centers for evaluation is required to consider other treatment modalities, including surgical resection of the seizure focus, vagus nerve stimulators, or implantable brain neurostimulators (4, 5). For individuals who are not suitable candidates for surgery, an alternative treatment modality such as the ketogenic diet (KD) can be considered (4, 6).

The KD is a high-fat, low-carbohydrate, and low-protein diet with restricted calories and fluids that mimic the metabolism of the fasting state to induce ketone bodies formation (7–9). Multiple processes that cause biochemical alterations are associated with KD, such as mediators responsible for neuronal hyperexcitability and cellular substrates (6). From a historical perspective, KD has been in clinical use since the early 1920s to control seizure in patients with DRE; Fasting and other dietary treatments have been in use to treat epilepsy since at least 500 BC (7, 10, 11). Furthermore, the Hippocratic collection recorded fasting as a therapeutic measure against epilepsy (12). There has been a notable resurgence in the global and regional adoption of such dietary treatments (10, 13–15). On this burgeoning list of diets, KD has received extensive interest owing to its beneficial effects, with emerging data supporting its use (16, 17). A recent systematic review of 14 randomized controlled trials (RCTs) that included 1,114 children to measure the efficacy of KD in DRE concluded that KD is an effective treatment modality for DRE in children and adolescents (18). Other data suggest a significantly positive outcome in using KD for DRE in children (19–22). Furthermore, within a more targeted context, a recent meta-analysis examined the effectiveness of different types of KD in the short term. The findings indicated that all evaluated diets played a significant role, showcasing distinct variations among the various treatment modalities (23).

Nonetheless, in terms of genetic epilepsy syndromes (GESs), very little data has been presented to assess the efficacy of KD. In one study, KD was particularly effective in patients with specific epilepsy-causing genetic mutations but lacked effectiveness among other epilepsy-causing mutations (24). Additionally, a prospective series of 57 children with genetic, structural, and metabolic abnormalities showed that KD could be an effective intervention for some patients with specific GESs, including Dravet syndrome, Lennox–Gastaut syndrome (LGS), and Wolf-Hirschhorn syndrome, which was responsive despite its highly refractory nature to all other therapies (25). Nevertheless, comprehensive data from larger cohorts can offer more conclusive insights into the suitability of the KD for patients with GESs. This study aims to emphasize the short-term effectiveness of the KD among pediatric patients diagnosed with GESs.

## Materials and methods

### Patient population and setting

This study involves a multi-center retrospective analysis of pediatric patients (<14 years) diagnosed with GESs and known genetic mutations. Genetic diagnosis was confirmed using next-generation sequencing (NGS) following adherence to appropriate ethical and logistical protocols. Neuroradiological investigations, including electroencephalography (EEG) and magnetic resonance imaging (MRI), were reviewed to assist with epilepsy syndrome diagnosis. EEG was performed in all but three patients. MRI was performed on the majority of patients for various clinical indications. The participating centers, located in the Gulf region, included patients from establishments in Saudi Arabia and Qatar to ensure sample diversity. Selection of centers was based on the availability of KD facilities and programs. Patients were classified according to syndrome and etiology utilizing the 1989 and more recent 2017 International League Against Epilepsy (ILAE) classification systems (26). The medical records of patients with DRE (defined as failure to respond to two or more ASMs) were retrospectively reviewed, and those meeting our eligibility criteria from the period between January and June 2024 were enrolled. The study adhered to strengthening the reporting of observational studies in epidemiology (STROBE) guidelines for retrospective studies (27).

### Study procedure and ketogenic protocol

**Figure 1:**
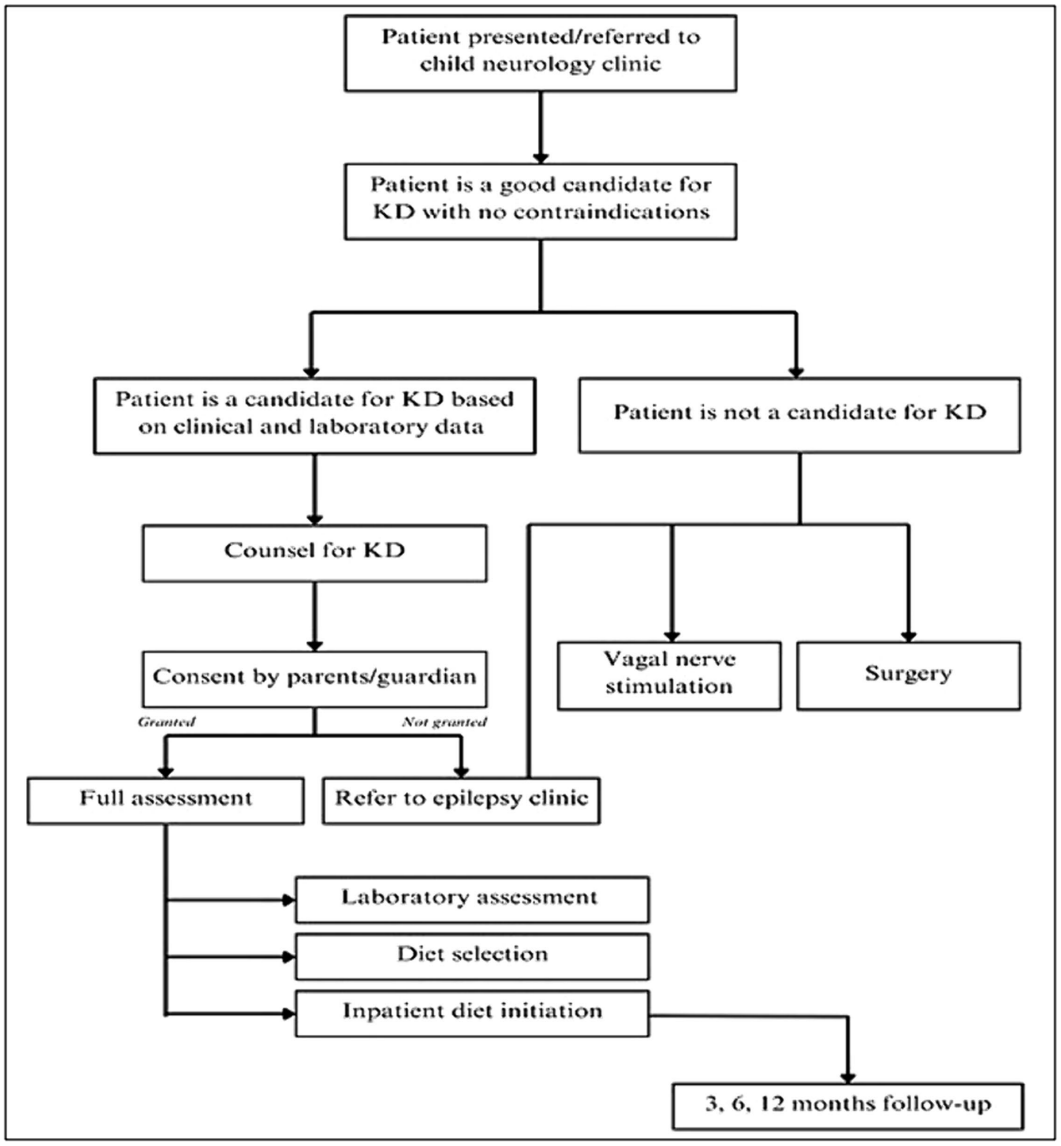
Algorithm of the keto-clinic protocol adopted in the study’s institutions.

Patients were recruited following the keto-clinic protocol (Figure 1), with exclusion criteria applied to those with absolute contraindications to the diet. The initiation of the KD was gradual, without fasting, for outpatients, except for cases where the patient is under 1 year of age, in critical condition, or the family requires training to monitor the diet’s response. Each patient’s dietary plan, based on their caloric requirements, was carefully crafted using KD food recipes combined with a ketogenic formula. The administration of KD was overseen by a multidisciplinary care team comprising pediatricians neurologists and licensed and specialized KD dietitians. Alongside KD as the primary treatment approach, participants received standard care provided to all patients in the ward. Two key parameters, baseline and prognostic data, were documented to assess the efficacy of KD among the participants. We categorized the effectiveness of KD in the patients into three categories: responders (≥50% seizure control), non-responders (<50% seizure control), and seizure freedom after a follow-up at 3, 6, and 12 months. Patients were labeled as responders whenever achieving ≥50% seizure control at any stage of the KD intervention. The genetic mutations included ion channels (calcium channels*: CACNA1E, CACNA1A, CACNA1H,* sodium channels*: SCN1A, SCN2A, SCN9A, SCN8A,* and potassium channels*: KCNQ2, KCNB1),* transporters and metabolism *(ABCA2, SLC2A1)* enzymes and enzyme modulators *(ATP6, ATP6V1A, AMT, PAFAH1B1, PPP3CA, PRUNE1, RARS2, ACADM),* structural and scaffold proteins *(ADAM22, ATL1, COL4A2, UNC80, WDR62),* receptors and signal transduction *(CNTNAP2, GRIN2A),* synaptic function and neurotransmission *(CREBBP, PIK3CA),* developmental and transcription regulators *(KIF1A, DOCK7, EXOC8, FGF12, FGF20, KDM5B, KMT2D, POGZ),* mitochondrial and metabolic disorders *(DNM1L, PDHA1, PEX3),* and miscellaneous genes *(STRADA, TSC1, TSC2, NCL Type 6, GLS, FAM111A, NR2FA)*. Clinical syndromes included LGS, Dravet syndrome, West syndrome, GLUT1 deficiency syndrome, tuberous sclerosis, Bosch-Boonstra-Schaaf syndrome, Congenital Lipomatous Overgrowth, Vascular Malformations, Epidermal Nevis, Spinal/Skeletal Anomalies/Scoliosis (CLOVES)/Megalencephaly-Capillary Malformation (MCM) syndrome, Angelman syndrome, Pitt-Hopkins-like syndrome-1, Landau–Kleffner syndrome, White Sutton syndrome, and unclassifiable encephalopathy with genetic mutations.

Genetic mutations in ion channels, such as *CACNA1E*, *CACNA1A*, and *CACNA1H* (calcium channels), as well as *SCN1A*, *SCN2A*, *SCN9A*, *SCN8A* (sodium channels), and *KCNQ2*, *KCNB1* (potassium channels), were associated with Dravet syndrome, West syndrome, and LGS. Mutations in transporters and metabolism genes including *ABCA2* and *SLC2A1* were associated with GLUT1 deficiency syndrome. Enzyme-related genes such as *ATP6*, *ATP6V1A*, *AMT*, *PAFAH1B1*, *PPP3CA*, *PRUNE1*, *RARS2*, and *ACADM* were associated with metabolic disorders and unclassifiable encephalopathy. Receptor and signal transduction mutations in *CNTNAP2* and *GRIN2A* were related to Landau–Kleffner syndrome and Pitt-Hopkins-like syndrome-1. Synaptic function and neurotransmission genes like *CREBBP* and *PIK3CA* were associated with CLOVES/MCM syndrome and Bosch-Boonstra-Schaaf syndrome. Developmental and transcription regulators such as *KIF1A*, *DOCK7*, *EXOC8*, *FGF12*, *FGF20*, *KDM5B*, *KMT2D*, and *POGZ* were implicated in syndromes like Angelman syndrome and White Sutton syndrome. Mitochondrial and metabolic disorder genes, including *DNM1L*, *PDHA1*, and *PEX3*, contributed to various unclassifiable encephalopathies. Lastly, miscellaneous genes like *STRADA*, *TSC1*, *TSC2*, *NCL* Type 6, *GLS*, *FAM111A*, and *NR2FA* were linked to conditions such as tuberous sclerosis and other genetic encephalopathies.

The assessment of KD compliance was conducted through dietary recall during follow-up visits. The primary outcome focused on changes in seizure frequency at 3, 6, and 12 months compared to the baseline before initiating the KD.

### Genetic testing and human sample collection

DNA capture probes were used alongside NGS-based copy number variation analysis with Illumina arrays. The coding regions of the gene, 10 bp of flanking intronic sequences, and known pathogenic/likely pathogenic variants within the gene (coding and non-coding) were targeted for analysis. Data analysis, variant calling, and annotation was performed using the Torrent Variant Caller software. The children’s variant inheritance mode was compared to the parents’ exome sequencing results upon availability of the data. Furthermore, after identifying a variant, the interpretation followed the 5-tier classification system recommended by the American College of Medical Genetics and Genomics and the Association for Molecular Pathology (28). According to the American College of Medical Genetics and Genomics and the Association for Molecular Pathology classification system, pathogenic or likely pathogenic variants were selected as causative mutations for epileptic activity (28).

### Instrument

The data collection instrument included various sections. The first included patients’ demographic data, which included gender, genetic and clinical diagnosis, current age, age of seizure onset, and the age of the KD initiation. The second section comprised the patients’ baseline clinical data, categorized based on the timing of KD initiation. The variables encompassed seizure etiology as per the ILAE Task Force guidelines for consistency in reporting, seizure frequency, characteristics, prior ineffective ASM treatments (before KD initiation), and the percentage of seizure control achieved. In the third section, the patients’ prognostic data were examined, focusing on the clinical outcomes post-KD initiation. This included assessing parameters such as seizure frequency, semiology, the number of ASMs utilized, and the evaluation of seizure control at 3, 6, and 12 months during follow-up visits. We also assessed the KD feeding route, patient’s adherence and reasons for discontinuation, and adverse events (if any) post-KD initiation. Also, the electrographic (i.e., EEG) and neuroradiological (i.e., MRI) data were collected upon availability for each patient and emphasized abnormal findings.

### Statistical analysis

The collected data was coded before the head, and IBM SPSS Statistics for Windows, version 27 (IBM Corp., Armonk, NY, USA) was used to analyze data. The sample size was not determined precisely because the subjects were *n* > 12, as previously suggested in the literature (29). In our sampling technique, we adopted Julious formulation (2005), which suggests a minimum sample size of 12 subjects per treatment arm (29). Categorical variables were represented using frequencies and percentages. For continuous variables, measures of central tendency were calculated. A paired samples *t*-test was employed to compare the mean seizure frequency before the start of the diet and 12 months after the start of the diet within each individual. Univariate binary logistic regression analysis was performed to identify factors significantly associated with the improvement of seizures. Potential confounders, including age at the time of the study, age at keto diet initiation, age at seizure onset, gender, seizure etiology, number of ASMs used, and route of feeding, were included in a multivariate regression model to adjust for their effects on the observed control in seizure frequency. Due to the adjuvant nature of KD in treating children with DRE, medication is unavoidable. As ASMs May be a potential factor influencing the outcome, we considered limiting such confounders in the statistical analysis. The results were presented as odds ratios (OR) with corresponding 95% confidence intervals (CI). A *p*-value of less than 0.05 was considered statistically significant.

## Results

### Sociodemographic and clinical characteristics

**Figure 2:**
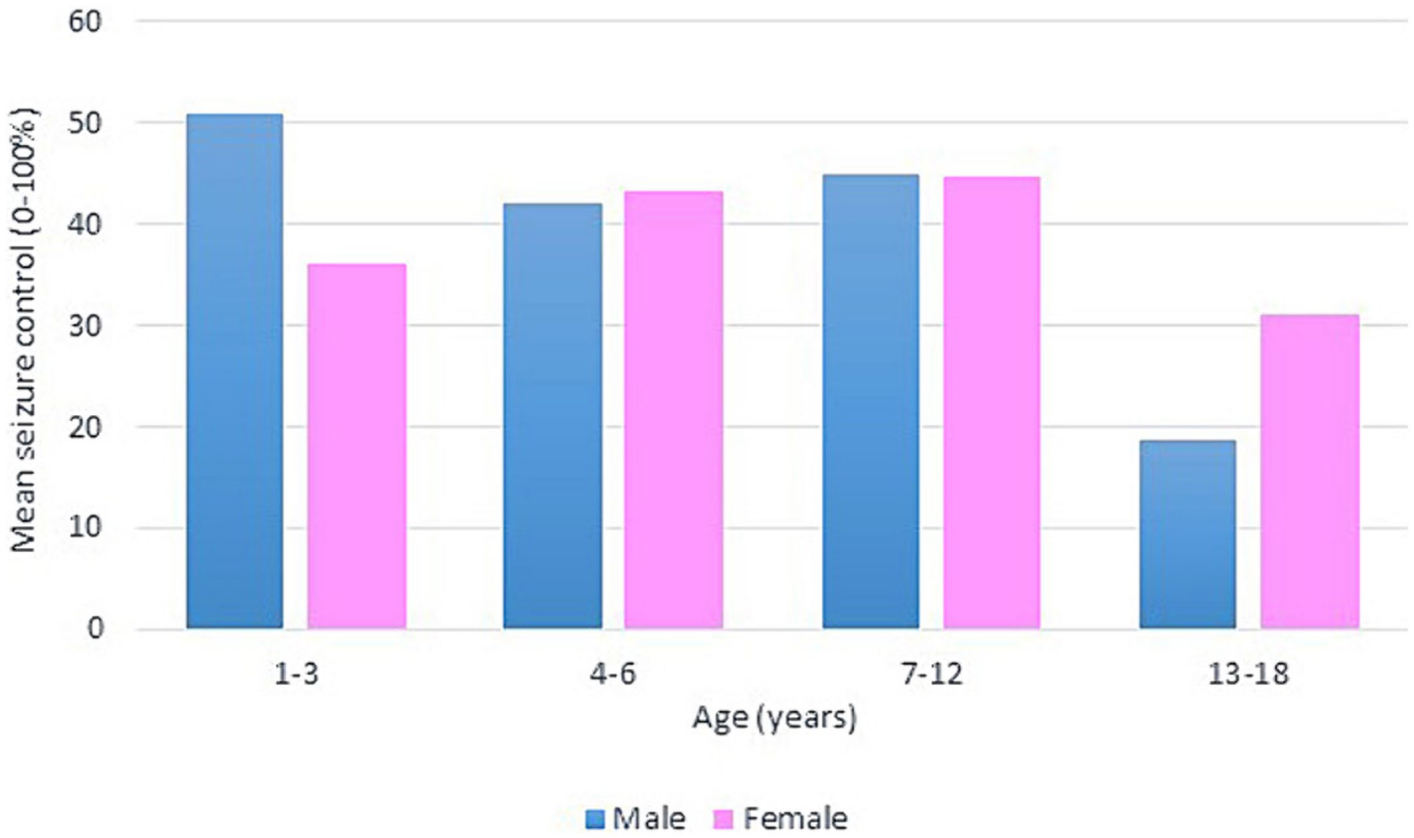
Bar chart of mean seizure control (0–100%) after 3 months of KD, by age group and gender.

**Figure 3:**
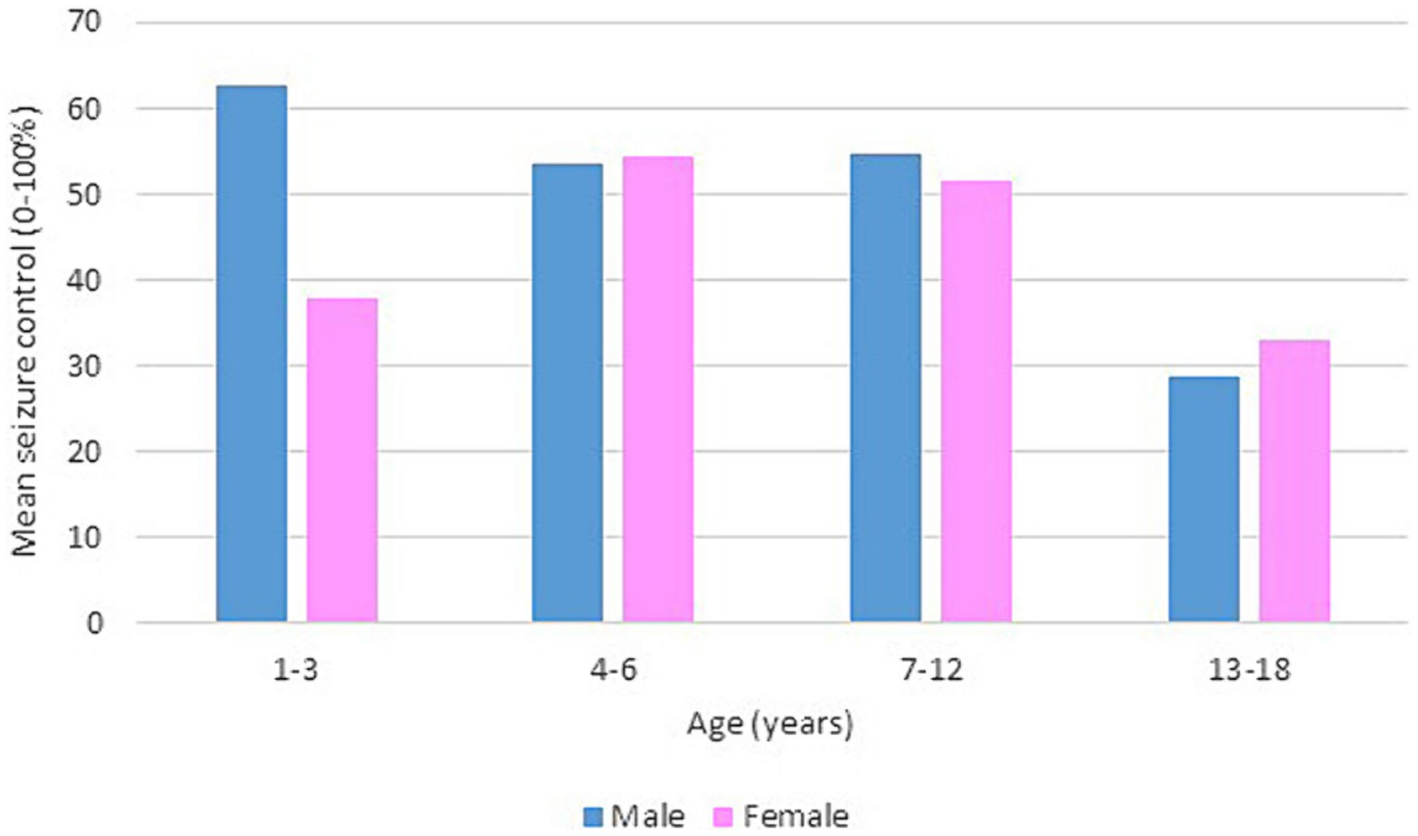
Bar chart of mean seizure control (0–100%) after 6 months of KD, by age group and gender.

**Figure 4:**
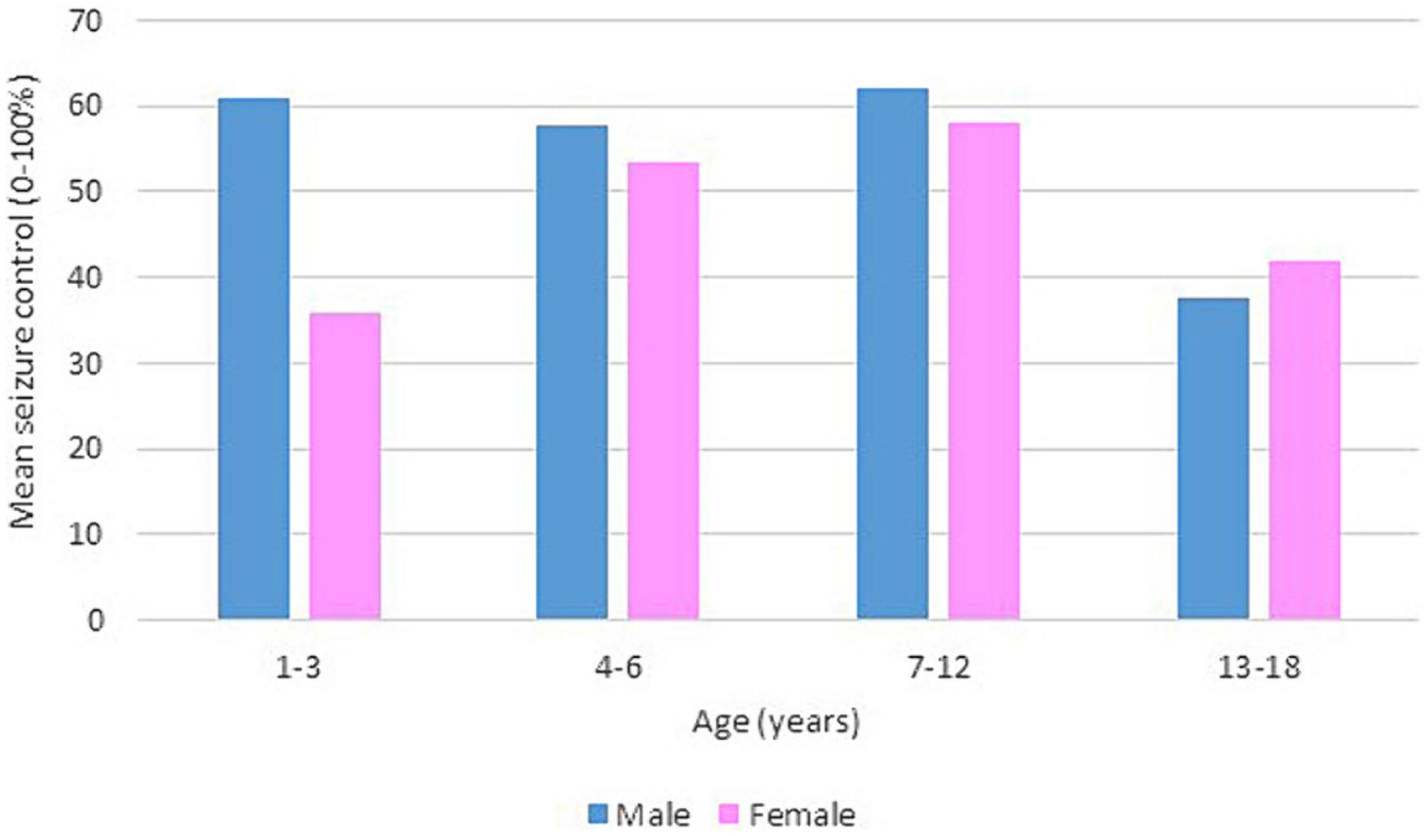
Bar chart of mean seizure control (0–100%) after 12 months of KD, by age group and gender.

Following our study procedure and protocols, 77 patients were included. Their mean current age is 7.94 ± 3.83 years. The group of responders had a mean current age of 7.58 ± 3.49 years, comprising 55 individuals, which accounted for 71.4% of the total. Notably, the mean age of seizure onset among responders was higher, at 22 months. In contrast, non-responders had a mean current age of 8.82 ± 4.53 years, with 22 individuals making up 28.6% of the group. The mean age of seizure onset for non-responders was 9 months, indicating an earlier onset. There was a slightly higher number of male patients in the study, totaling 42 individuals and representing 54.6% of the total study cohort. Females constituted 35 patients, comprising of 45.5%. In terms of respondents, 30 male patients achieved seizure improvement (≥50 seizure control), comprising 71.4% of the total male patients. There were 25 female patients achieving improvement and 10 with no improvement. The majority of our patients experienced monthly seizures after the start of the diet; of those, 89.3% achieved seizure improvement with the diet and had been experiencing either daily or weekly episodes of seizure prior to the initiation of the diet. Out of the 22 patients with weekly seizures, 59.1% showed improvement in their seizure frequency following the diet. Patients who experienced daily seizures both before and after the dietary intervention had the highest rate of lack of improvement (66.7%), with their seizure frequency remaining unchanged despite the diet. Interestingly, 33.3% of these patients who continued to have daily seizures did report reduction or control in their seizure activity. An inverse relationship was noted between the number of antiseizure medications (ASMs) used and the rate of seizures improvement. Patients who did not use any ASMs or were on a single medication showed a 100% improvement rate on the dietary intervention. Among those on two medications, 80.0% experienced improvement, while patients using three or more medications achieved improvement in 63.0% of cases. We observed a 70.3% improvement in patients with abnormal EEG results, while 73.2% of those with abnormal brain MRI findings showed improvement with the dietary intervention. In Table 1, detailed information on improvement rates based on clinical data are presented (Table 1). The trend of seizure control based on the gender of our patients across the three intervals of our study is demonstrated in Figures 2–4.

**Table 1:** Sociodemographic and clinical characteristics of children on KD: improved vs. not-improved seizure outcomes (*n* = 77).

Characteristics	Seizure outcome after KD	
	Improved (*n* = 55, 71.4%)	Not-improved (*n* = 22, 28.6%)
Current age (years)	7.58 ± 3.49	8.82 ± 4.53
Age of KD initiation (years)	4.72 ± 3.16	4.94 ± 3.95
Age of seizure onset (months), median (IQR)	7 (22)	5 (9)
Gender		
Male	30 (71.4)	12 (28.6)
Female	25 (71.4)	10 (28.6)
Seizure frequency		
Daily	4 (33.3)	8 (66.7)
Weekly	13 (59.1)	9 (40.9)
Monthly	25 (89.3)	3 (10.7)
Controlled	13 (86.7)	2 (13.3)
† Number of ASMs used		
None	2 (100.0)	0 (0.0)
1	2 (100.0)	0 (0.0)
2	16 (80.0)	4 (20.0)
≥3	29 (63.0)	17 (37.0)
Route of feeding		
Gastric tube (GT)	23 (67.6)	11 (32.4)
Oral	32 (74.4)	11 (25.6)
Number of reported adverse effects		
None	32 (76.2)	10 (23.8)
1	12 (66.7)	6 (33.3)
2	10 (71.4)	4 (28.6)
≥3	1 (33.3)	2 (66.7)
Abnormal EEG	52 (70.3)	22 (29.7)
Abnormal MRI	41 (73.2)	15 (26.8)

KD, ketogenic diet. EEG, electroencephalogram; and MRI, magnetic resonance imaging. Data are presented as number (%) or mean ± SD. †, Data was available for *N* = 70; SD, standard deviation; IQR, interquartile range.

### Seizure outcome based on etiology and diagnoses

As to the etiology of the seizure, patients were designated into on the following groups; genetic, structural, metabolic disorder, immune disorder, infectious, or unknown causes. The genetic etiology group had the largest number of patients, with individuals in this group representing 78.8% of the total. Patients with structural etiology showed improvement in 66.7% of the cases, while individuals with metabolic and unknown etiologies experienced a higher rate of no improvement compared to those who showed improvement (Table 2).

**Table 2:** Clinical diagnosis and seizure etiology of children on KD: improved vs. not-improved seizure outcomes (*n* = 77).

Characteristics	Seizure outcome after KD	
	Improved (*n* = 55, 71.4%)	Not-improved (*n* = 22, 28.6%)
Clinical diagnosis		
Lennox–Gastaut syndrome	14 (93.3)	1 (6.7)
Unclassifiable encephalopathies	11 (78.6)	3 (21.4)
Dravet syndrome	6 (75.0)	2 (25.0)
West syndrome	3 (75.0)	1 (25.0)
GLUT1 deficiency syndrome	2 (50.0)	2 (50.0)
Tuberous sclerosis	2 (100.0)	0 (0.0)
Bosch-Boonstera-Schaaf syndrome	1 (100.0)	0 (0.0)
CLOVES/MCM syndrome	1 (100.0)	0 (0.0)
Angelman syndrome	0 (0.0)	1 (100.0)
Pitt-Hopkins-like syndrome-1	1 (100.0)	0 (0.0)
Landau–Kleffner syndrome	1 (100.0)	0 (0.0)
White Sutton syndrome	1 (100.0)	0 (0.0)
Seizure etiology		
Genetic	41 (78.8)	11 (21.2)
Structural	6 (66.7)	3 (33.3)
Metabolic disorder	0 (0.0)	1 (100.0)
Immune disorder	1 (100.0)	0 (0.0)
Infectious	4 (80.0)	1 (20.0)
Unknown	3 (33.3)	6 (66.7)

KD, ketogenic diet. GLUT1, Glucose Transporter Type 1; ASMs, anti-seizure medications; EEG, electroencephalogram; MRI, Magnetic resonance imaging. CLOVES/MCM, Congenital Lipomatous Overgrowth, Vascular Malformations, Epidermal Nevis, Spinal/Skeletal Anomalies/Scoliosis/Megalencephaly-Capillary Malformation.

Regarding the clinical diagnosis, the majority of patients were diagnosed with LGS (*n* = 15, 19.5%), followed by unclassifiable encephalopathies (*n* = 14, 18.1%), Dravet syndrome (*n* = 8, 10.4%), West syndrome (*n* = 4, 5.2%), glucose transporter type 1 (GLUT1) deficiency syndrome (*n* = 4, 5.2%), and others described in Table 2. Regarding improvements to the diet, 93.3% of patients diagnosed with LGS achieved seizure improvement at a specific interval (i.e., 3, 6, or 12 months) of their diet intake. While patients with unclassifiable encephalopathies have a 78.6% improvement, followed closely by patients with Dravet syndrome who had improvement among 75.0% of the patients. Among the conditions where 100.0% of the patients showed improvement were tuberous sclerosis, Bosch-Boonstera-Schaaf syndrome, CLOVES/MCM syndrome, Pitt-Hopkins-like syndrome-1, Landau–Kleffner syndrome, and White Sutton syndrome. However, we only recruited one patient for each condition except for tuberous sclerosis. Other diagnoses are described in Table 2.

### Genetic profile and diet efficacy

When evaluating patient responses based on their genetic background, notable findings emerged, indicating that individuals with LGS exhibited the most significant improvement in seizure control. The assessment was conducted at 3, 6, and 12-month intervals. Initially, 12 out of 15 patients demonstrated improved seizure control during the first interval, as outlined in the methodology. Subsequently, one more patient showed improvement in the second interval, followed by another patient in the third interval. Throughout our observation period, the total number of respondents who experienced improved seizures within the LGS group reached 14 out of 15 patients.

The genetic mutations observed in these patients included *SCN8A*, *CACNA1H*, and *KIF1A*. In the initial interval, 5 out of 8 patients with Dravet syndrome were classified as responders, a proportion that remained consistent in the subsequent two intervals, with 6 out of 8 patients showing positive response to treatment. The primary genetic mutations identified in Dravet syndrome cases were *SCN1A*, followed by *SCN2A* and *SCN9A*. Within the cohort of four patients diagnosed with West syndrome, initially two patients responded to treatment, with a third patient showing a positive response in the subsequent interval. Among the responders with West syndrome, three out of four patients were identified, with mutations detected in *EXOC8* and *PEX3* genes. In patients with GLUT1 deficiency syndrome, two individuals displayed a positive response without any increase in the number of responders in the following months. More detailed information on other conditions can be found in Table 3.

**Table 3:** Number of responders by genetic diagnosis at 3, 6, and 12 months.

Genetic diagnosis	No. of responders at 3 months	No. of responders at 6 months	No. of responders at 12 months
Lennox–Gastaut syndrome	12/15	13/15	14/15
Dravet syndrome	5/8	6/8	6/8
West syndrome	2/4	3/4	3/4
GLUT1 deficiency syndrome	2/4	2/4	2/4
Tuberous sclerosis	1/2	2/2	2/2
Bosch-Boonstera-Schaaf syndrome	0/1	1/1	1/1
CLOVES/MCM syndrome	1/1	1/1	1/1
Angelman syndrome	0/1	0/1	0/1
Pitt-Hopkins-like syndrome-1	0/1	1/1	1/1
Landau–Kleffner syndrome	1/1	1/1	1/1
White Sutton syndrome	0/1	1/1	1/1

No., Number; LGS, Lennox–Gastaut syndrome; and GLUT1, glucose transporter type 1. CLOVES/MCM, Congenital Lipomatous Overgrowth, Vascular Malformations, Epidermal Nevis, Spinal/Skeletal Anomalies/Scoliosis/Megalencephaly-Capillary Malformation.

### Prognostic data

We assessed seizure control across different etiologies in Table 4. A significant *p*-value was observed in patients with genetic and structural etiologies, *p* < 0.001 and *p* < 0.047, respectively. This indicates a positive response to the dietary intervention among patients with these two etiologies. In detail, the mean number of seizures in patients with genetic etiology prior to the diet was 18.752 ± 19.80 percent. Which notably rose to 60.77 ± 26.10 percent after initiating the diet. Similarly, those with structural etiology, had a mean control of 22.00 ± 23.48 percent before the diet, increasing to 55.00 ± 35.43 percent following the diet. Patients with infectious and unknown etiologies did not show significant improvement following the dietary intervention. Specifically, individuals with unknown etiology had mean control rates of 13.33 ± 9.35 percent before the diet initiation, increasing to 30.00 ± 28.72 percent after the diet (*p* = 0.092). Similarly, patients with infectious etiology displayed mean control rates of 11.00 ± 11.40 percent before the diet, slightly rising to 47.00 ± 30.94 percent after the diet (*p* = 0.102). The analysis of the KD’s impact on various genetic mutations revealed varying levels of improvement across gene groups. Although most groups exhibited some degree of improvement, none reached statistical significance (Table 5). Specifically, 83.3% of patients with ion channel mutations and all patients in the miscellaneous genes group showed improvement. Similarly, both the receptors and signal transduction, as well as the synaptic function and neurotransmission groups, exhibited 100% improvement, though these groups had limited sample sizes. The enzymes and enzyme modulators group showed a non-significant trend toward fewer improvements (42.9%, *p* = 0.075).

**Table 4:** Seizure control rate before and after 12 months of the start of KD according to seizure etiology.

Seizure etiology	Seizure control (0–100%) before KD	Seizure control (0–100%) after 12 months of KD	*p-value*
Genetic	18.752 ± 19.80	60.77 ± 26.10	<0.001*
Structural	22.00 ± 23.48	55.00 ± 35.43	<0.047*
Unknown	13.33 ± 9.35	30.00 ± 28.72	0.092
Infectious	11.00 ± 11.40	47.00 ± 30.94	0.102

KD, ketogenic diet. Data are presented as mean ± SD. SD, standard deviation. *P-value is significant at *p* < 0.05.

**Table 5:** Impact of the KD on patient outcomes across various genetic mutations.

Gene group	Improved	Not-improved	*P*-value
Ion channels	10 (83.3)	2 (16.7)	0.469
Transporters and metabolism	2 (50)	2 (50)	0.294
Enzymes and enzyme modulators	3 (42.9)	4 (57.1)	0.075
Structural and scaffold proteins	3 (60)	2 (40)	0.602
Receptors and signal transduction	1 (100)	0 (0)	1.000
Synaptic function and neurotransmission	1 (100)	0 (0)	1.000
Developmental and transcription regulators	5 (71.4)	2 (28.6)	1.000
Mitochondrial and metabolic disorders	2 (66.7)	1 (33.3)	1.000
Miscellaneous genes	8 (100)	0 (0)	0.088

KD, ketogenic diet. Data are presented as number (%).

### Evaluation of the efficacy and adverse events

We conducted a univariate and multivariate logistic regression analysis of factors contributing to seizure control (Table 6). Among the independent variables were their current age, age of KD initiation, age of seizure onset, male gender, seizures of genetic etiology, the number of used ASMs grouped into patients with none, one, or two medications into one group and patients with three or more medications, and oral feeding as a route of feeding. The groups with a significant seizure control after the diet using the univariate logistic regression included those with seizures of genetic etiology (*p* = 0.041; OR, 2.929; CI 95%, 1.043–8.226). After adjusting the model for common confounders (age at the time of the study, age of KD initiation, age of seizure onset, gender, seizure etiology, number of ASMs, and route of feeding), we found two independent factors significantly affecting the seizure control. They included seizures of genetic etiology (*p* = 0.021; OR, 5.633; CI 95%, 1.294–24.531) and the number of used ASMs (*p* = 0.037; OR, 0.217; CI 95%, 0.052–0.911). There was an inverse relationship between the number of ASMs used and the response to the dietary intervention, with patients taking three or more ASMs showing a higher likelihood of a suboptimal response. Among the 35 patients who reported adverse events, 18 experienced a single adverse event, 14 had two types of adverse events, and three reported three adverse events. The most commonly reported adverse event was constipation (*n* = 20, 26.0%), followed by irritability (*n* = 11, 14.3%) and nausea (*n* = 7, 9.1%). Additional reported events included vomiting, weight loss, fatigue, hypoglycemia, nutritional deficiencies, kidney stones, and pancreatitis.

**Table 6:** Univariate and multivariate logistic regression analysis of factors contributing to seizure control.

Independent variables	Univariate logistic regression			Multivariate logistic regression		
	*P*-value	Crude OR	95% CI of OR	*P*-value	†Adjusted OR	95% CI of OR
Current age (numerical)	0.202	0.917	0.802–1.048	0.243	0.858	0.664–1.109
Age of KD initiation (numerical)	0.788	0.980	0.846–1.135	0.733	1.049	0.797–1.382
Age of seizure onset (numerical)	0.212	1.024	0.987–1.062	0.101	1.039	0.992–1.088
Male gender	1.000	1.000	0.370–2.699	0.406	0.603	0.183–1.991
Seizures of genetic etiology	0.041*	2.929	1.043–8.226	0.021*	5.633	1.294–24.531
Using 3 or more ASMs	0.053	0.328	0.106–1.014	0.037*	0.217	0.052–0.911
Route of feeding (oral)	0.514	1.391	0.516–3.755	0.162	3.413	0.610–19.088

KD, ketogenic diet. ASMs, anti-seizure medications. Adjusted OR†, model adjusted for common confounders (age at the time of the study, age of keto diet initiation, age of seizure onset, gender, seizure etiology, number of ASMs, and route of feeding); *P-value is significant at *P* < 0.05. CI, confidence interval; OR, odds ratio; ASMs, anti-seizure medications.

## Discussion

To the best of our knowledge, this is the first study conducted in the Arab region to evaluate the effectiveness and tolerability of the KD among patients with GESs. This research focused on analyzing seizure outcomes concerning socio-clinical determinants and diet-related factors. The study encompassed patients from six centers across Saudi Arabia and Qatar, diagnosed with a variety of conditions, such as LGS, Dravet syndrome, West syndrome, GLUT1 deficiency syndrome, tuberous sclerosis, Bosch-Boonstra-Schaaf syndrome, CLOVES/MCM syndrome, Angelman syndrome, Pitt-Hopkins-like syndrome-1, Landau–Kleffner syndrome, and White Sutton syndrome.

Our findings revealed that younger patients exhibited a more positive response to the KD compared to older patients, regardless of their clinical characteristic or the age at which they began the diet. The overall improvement rate, assessed using seizure reduction, among patients after initiating the diet was notably high at 71.43%. This is higher than previously reported studies which suggest 44–66.7% seizure reduction among children with DREs (30, 31).

Additionally, patients who responded favorably to the KD tended to have a later onset of their first seizure episode. This observation is consistent with existing knowledge that an earlier onset of seizures is generally associated with poorer outcomes (32). Our study supports the view that the timing of seizure onset and patient age are crucial factors in predicting the effectiveness of dietary interventions in managing GESs.

Our analysis did not find a significant association between gender and response to the KD. However, gender differences in seizure susceptibility and other epileptogenic processes have been reported in the literature, with males often exhibiting greater seizure susceptibility than females (33, 34). In our study, gender appeared to be a confounding factor that, in combination with other clinical variables, that May contribute to seizure control.

After controlling for potential confounders such as gender, current age, age of seizure onset, age of KD initiation, seizure etiology, number of ASMs used, and route of feeding, two variables—genetic etiology and the number of ASMs used—were significantly associated with higher seizure control (see Table 6). This aligns with findings by Ko et al., who reported the efficacy of KD among GESs, specifically in patients with *SCN1A*, *KCNQ2*, *STXBP1*, and *SCN2A* mutations, though less effective in those with *CDKL5* mutations (24). Similarly, we observed seizure control in specific GESs, including Dravet syndrome (mainly caused by *SCN1A* mutations) and LGS, which can involve mutations in *CDKL5*, *DNM1*, *STXBP1*, or *SCN2A* genes. A meta-analysis by Wang et al. further supports KD’s use in GESs, particularly Dravet syndrome (35). However, our sub-analysis of the genetic mutation revealed no statistically significant relationship between specific mutations and seizure improvement after initiating the KD. Nonetheless, this could be attributed to the small number of patients carrying each mutation whereas a larger sample can potentially lead to statistical association. In Table 7, we summarized the literature findings regarding the effectiveness of KD in genetic epilepsies and certain genetic mutations (24, 36–45).

**Table 7:** Literature studies investigating the efficacy of KD in genetic epilepsies.

Author	Year	Design	Sample size	Number of responder	Genes with significant improvement
Song et al. (36)	2024	Retrospective	32	26 (at 12 months)	*ACTL6B, ARX, GABRB2, NF1, PIGA, SCN8A, SPTAN1, STXBP1, and RHOBTB2*
Nam et al. (37)	2022	Retrospective	12	2 (at 12 months)	*STXBP1*
Ko et al. (24)	2018	Retrospective	73	32 (at 12 months)	*SCN2A, STXBP1, KCNQ2, and SCN1A*
Wiemer-Kruel (38)	2017	Retrospective	30	25 (at 42 months)	*–*
Mori et al. (39)	2016	Case report	1	1 (at 9 month)	*KCNT1*
Müller et al. (40)	2016	Retrospective	38	9 (at 12 months)	*CDKL5*
Dressler et al. (41)	2015	Retrospective	10	6 (at 12 months)	*SCN1A*
Lemmon et al., (42)	2012	Retrospective	71	36 (at 12 months)	–
Caraballo (43)	2011	Retrospective	24	16 (at 24 months)	–
Nabbout et al. (44)	2011	Prospective	15	5 (at 12 months)	–
Liebhaber et al. (45)	2003	Case report	1	1 (at 12 months)	*MECP2*

KD, ketogenic diet.

Similarly, our study noted a favorable response to KD in 75% of patients with West syndrome at 1 year, consistent with prior observations. For instance, Ko et al. found KD to be more effective in patients with West syndrome lacking *CDKL5* mutations when compared to those with the mutation (24).

Comparing seizure rates before and after KD administration, we observed a significant control in seizures among patients with genetic (*p* < 0.001) and structural etiologies (*p* < 0.047). This supports other studies’ findings suggesting KD’s efficacy across various etiologies, including developmental and epileptic encephalopathy due to acquired structural etiologies (46). Although surprising, growing literature echoes these results (47).

Another significant factor affecting the response to KD was the number of ASMs used. Children who had tried fewer ASMs before starting KD showed better responses. This is consistent with the literature, indicating that children who have failed multiple ASMs tend to respond less favorably to KD. This could be attributed to the fact that children categorized as having DRE often present with more severe and refractory forms of epilepsy, suggesting a more complex underlying pathophysiology (48, 49).

Like many epilepsy treatments, the KD is associated with adverse events. In our cohort, we observed various adverse events following KD initiation. Specifically, 18 patients experienced one type of adverse event, 14 reported two events, and three reported three adverse events. Despite these occurrences, the overall safety profile of the diet was considered acceptable, as the majority of adverse events being mild in nature. Common adverse effects included constipation, irritability, nausea, vomiting, nutritional deficiencies, hypoglycemia, pancreatitis, and kidney stones. Gastrointestinal issues were the leading complaints, consistent with findings in the literature (50). Vomiting was reported in some of our patients, and previous reports suggest that this complication can affect approximately one-fourth of cases (51). Kidney stones, another significant concern, can be observed in 3–10% of patients (52). This risk can be mitigated through adequate hydration, using oral citrates, and avoiding certain ASMs known to increase the likelihood of stone formation, such as zonisamide, and acetazolamide (53, 54).

The study was limited to a lack of studies nationwide for comparative analysis. Small sample sizes in certain clinical diagnoses prevented definitive conclusions about the diet’s efficacy in these groups. Furthermore, the lack of sufficient number of participants in certain groups including the low number of patients with metabolic and infectious etiologies in Table 2 compared to other etiologies May have impacted the outcome of the related analysis. Recall bias May have skewed reported responses to the diet. The concurrent use of ASMs could impact patient outcomes and responses to the diet, prompting us to regard ASMs as a potential confounding factor in the logistic regression model. To address these issues, we increased our sample size and recruited patients from multiple centers.

## Conclusion

This study provides the first comprehensive evaluation of the KD for patients with GESs in the Arab region. Our findings indicate a significant improvement in seizure control, especially among younger patients and those with a later onset of seizures. This highlights the importance of early intervention and age in predicting the success of dietary treatments for GESs. Patients with specific genetic mutations responded well to KD, aligning with previous research, though efficacy varied across genetic profiles. Additionally, the ASMs used before starting KD is crucial in predicting response. Therefore, selecting appropriate candidates is vital, particularly in resource-limited settings.

Our study confirms significant seizure control improvements in patients with genetic and structural etiologies, supporting the broad applicability of KD across various epileptic conditions. KD emerges as an effective treatment option for GESs, advocating its consideration as a viable strategy for managing DRE. This underscores the need for ongoing research to optimize and personalize dietary interventions based on genetic and clinical profiles. Further research is necessary to explore the long-term benefits and potential side effects of KD and to identify additional socio-clinical determinants influencing treatment outcomes. Such efforts are essential for enhancing our understanding of the optimal use of KD in managing genetic epileptic syndromes.

## Data Availability

The original contributions presented in the study are included in the article/supplementary material, further inquiries can be directed to the corresponding author/s.
